# Avocado oil alleviates non-alcoholic fatty liver disease by improving mitochondrial function, oxidative stress and inflammation in rats fed a high fat–High fructose diet

**DOI:** 10.3389/fphar.2022.1089130

**Published:** 2022-12-19

**Authors:** Claudia Isabel García-Berumen, Manuel Alejandro Vargas-Vargas, Omar Ortiz-Avila, Rosa María Piña–Zentella, Minerva Ramos-Gómez, María del Consuelo Figueroa–García, Ricardo Mejía-Zepeda, Alain Raimundo Rodríguez–Orozco, Alfredo Saavedra–Molina, Christian Cortés-Rojo

**Affiliations:** ^1^ Instituto de Investigaciones Químico–Biológicas, Universidad Michoacana de San Nicolás de Hidalgo, Morelia, México; ^2^ Facultad de Enfermería, Universidad Michoacana de San Nicolás de Hidalgo, Morelia, México; ^3^ Facultad de Química, Universidad Autónoma de Querétaro, Querétaro, México; ^4^ Unidad de Biomedicina, Facultad de Estudios Superiores Iztacala, Universidad Nacional Autónoma de México, Tlalnepantla de Baz, México; ^5^ Facultad de Ciencias Médicas y Biológicas “Dr. Ignacio Chávez”, Universidad Michoacana de San Nicolás de Hidalgo, Morelia, México

**Keywords:** NAFLD, mitochondria, ROS, hyperglycemia, proinflammatory cytokines, Persea americana Mill.

## Abstract

Non-alcoholic fatty liver disease (NAFLD) is characterized by lipid accumulation in hepatocytes, and in advanced stages, by inflammation and fibrosis. Excessive ROS production due to mitochondrial dysfunction contributes to NAFLD development, making the decrease in mitochondrial ROS production an emerging target to alleviate NAFLD. Previously, we have shown that avocado oil, a source of several bioactive compounds with antioxidant effects, decreases oxidative stress by improving the function of the mitochondrial electron transport chain (ETC) and decreasing ROS levels in mitochondria of diabetic and hypertensive rats. Therefore, we tested in this work whether avocado oil alleviates NAFLD by attenuating mitochondrial dysfunction, oxidative stress and inflammation. NAFLD was induced in rats by a high fat—high fructose (HF) diet administered for six (HF6) or twelve (HF12) weeks. Hepatic steatosis, hypertrophy and inflammation were detected in both the HF6 and HF12 groups. Hyperglycemia was observed only in the HF12 group. The HF6 and HF12 groups displayed dyslipidemia, impairments in mitochondrial respiration, complex III activity, and electron transfer in cytochromes in the complex III. This led to an increase in the levels of ROS and lipid peroxidation. The substitution of the HF6 diet by standard chow and avocado oil for 6 weeks (HF6+AVO + D), or supplementation of the HF12 diet with avocado oil (HF12 + AVO), ameliorated NAFLD, hyperglycemia, dyslipidemia, and counteracted mitochondrial dysfunctions and oxidative stress. The substitution of the HF6 diet by standard chow without avocado oil did not correct many of these abnormalities, confirming that the removal of the HF diet is not enough to counteract NAFLD and mitochondrial dysfunction. In summary, avocado oil decreases NAFLD by improving mitochondrial function, oxidative stress, and inflammation.

## 1 Introduction

Non-alcoholic fatty liver disease (NAFLD) comprises a spectrum of liver alterations ranging from simple steatosis, steatohepatitis, to cirrhosis. NAFLD is one of the most prevalent liver diseases in the world. However, no drug has been approved for NAFLD treatment so far (Tacelli et al., 2018). This has urged the search for novel strategies to attenuate NAFLD progression.

Both mitochondrial dysfunction and oxidative stress may be targeted to alleviate NAFLD, as they are hallmarks of NAFLD development (Simões et al., 2018). Mitochondrial alterations during NAFLD include enhanced mitochondrial fission, impaired oxidative phosphorylation, decreased activity of the electron transport chain (ETC) complexes, and oxidative stress (Galloway et al., 2014). The latter has been involved in increased activity of pro-inflammatory cytokines that promote disease progression to steatohepatitis and cirrhosis (Bellanti et al., 2017).

The fruit of the avocado tree (*Persea americana Mill.*) is a source of an oil with remarkable biological effects that may be useful in the treatment of NAFLD. The main fatty acid in avocado oil is oleic acid (C18:1) independently of the avocado variety and the country of source (Flores, et al., 2019). β-carotene has been identified as the most abundant tocopherol in avocado oil (Flores, et al., 2014). A variety of phytosterols has also been identified in avocado oil, being β-sitosterol the most abundant and in a higher amount than in olive oil (Berasategi, et al., 2012). In a study with overweight humans consuming for 6 days a high-fat–hypercaloric diet where butter was substituted by avocado oil, it was found an improvement in postprandial serum lipid profile, glycemia, and insulin levels, besides a decrease in serum markers of inflammation (Furlan, et al., 2017).

A characterization of avocado oil effects on mitochondrial function and oxidative stress has been carried out in preclinical model of diseases. In diabetic rats, we have shown that avocado oil improves the activity of the ETC in liver, brain and kidney mitochondria. This led to lower mitochondrial levels of ROS and oxidative stress (Ortiz-Avila et al., 2015a; 2015b, 2013). In the kidneys of hypertensive rats, avocado oil prevented renal damage, mitochondrial dysfunction and oxidative stress, and improved renal vascular function (Márquez-Ramírez, et al., 2021). These data support further research of avocado oil as a natural product regulating mitochondrial dysfunction in diseases like NAFLD. On this basis and given the beneficial effect of avocado oil on systemic markers of metabolism and inflammation in humans (Furlan, et al., 2017), we have explored whether avocado oil improves NAFLD, mitochondrial function, oxidative stress, and inflammation. We also tested if avocado oil counteracts these alterations when it is given once NAFLD was established or if it is effective only when administered along with a high fat—high fructose diet during NAFLD development.

## 2 Materials and methods

### 2.1 Animals and diets

Male Wistar rats weighing ∼330 g were used and kept in a bioterium with controlled temperature and light/dark cycles of 12 h/12 h. Each rat was maintained in individual cages. The animals were managed according to the recommendations from Mexican Federal Regulations for the Use and Care of Animals (NOM-062-ZOO-1999) by the Ministry of Agriculture, Mexico. This research was approved by the Institutional Bioethics and Biosecurity Committee of the Instituto de Investigaciones Químico-Biológicas, Universidad Michoacana de San Nicolás de Hidalgo.

**FIGURE 1:**
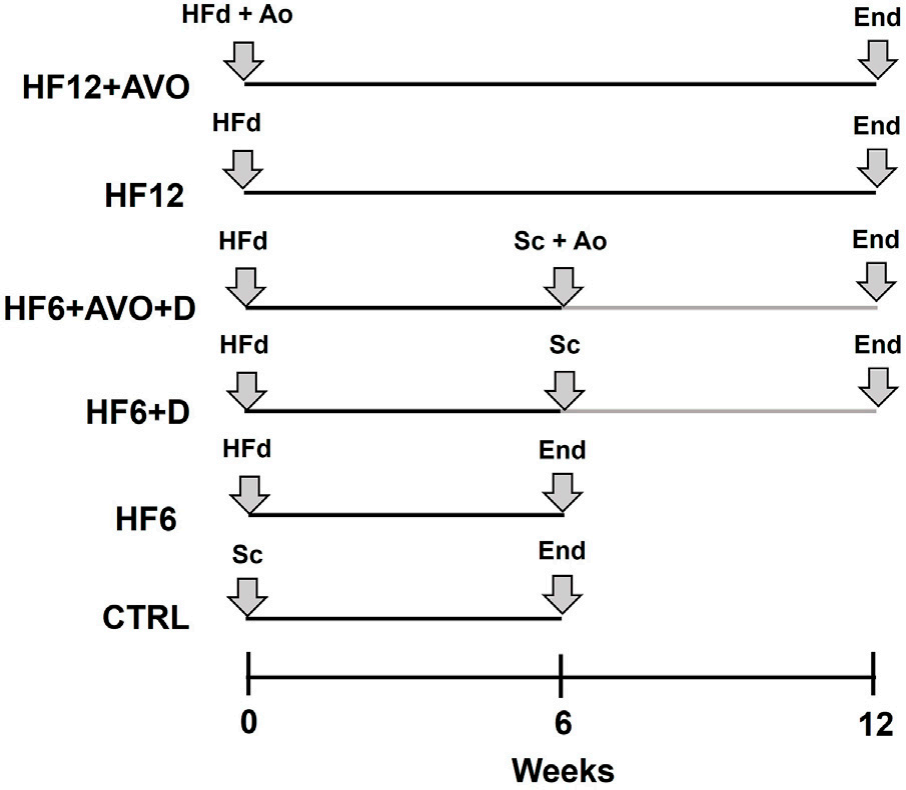
Graphical description of the time course of the experimental diets. CTRL: rats were fed for 6 weeks with standard chow; HF6: the HF diet was given for 6 weeks; HF12: the HF diet was given for 12 weeks; HF6+D: the HF diet was given for 6 weeks and then, it was substituted by standard chow and given for additional 6 weeks; HF6+AVO + D: the HF diet was given for 6 weeks and then, it was substituted by standard chow plus avocado oil and given for additional 6 weeks; HF12 + AVO: the HF12 diet was given for 12 weeks supplemented with avocado oil. Sc: standard chow; End: end of the experimental period at which the rats were sacrificed; HFd: high fat - high fructose diet; Ao: avocado oil.

NAFLD was induced by a high fat diet prepared by mixing equal quantities of standard rodent chow (Laboratory Rodent Diet 5001, LabDiet, St. Louis, MO, United States) and a dough prepared with 20% hydrogenated vegetable oil, 20% sucrose, 20% lactose, 5% lard, 2% sodium cholate, 0.4% choline chloride and 0.15% thiouracil (Table 1). The diet was prepared every week in our laboratory and stored at 4°C. Twenty-5 g of this diet was administered daily at the time periods indicated in Figure 1. When indicated, 200 ml fructose (25% v/v) was included in the drinking water and given to each rat every other day. Avocado oil was orally administered by gavage at a dose of 1 ml/250 g weight, using a commercial presentation of avocado oil (Ahuacatlan, DIRICOM, S.A. de C.V., México), purchased from a local grocery. This oil contains 0.2 g/ml of saturated fatty acids, 0.2 g/ml of polyunsaturated fatty acids, and 0.6 g/ml of monounsaturated fatty acids.

**TABLE 1:** Experimental groups and preparation of diets.

Diet components	Experimental diets		
	Control	High fat—high fructose (HF)	High fat—high fructose plus avocado oil (HF + AVO)
Calories in 100 g	336	423.25	435.05
Standard rodent chow^a^,%	100	50	50
Total fat,%	13.49	20.01	21.01
-Saturated fat,%	1.56	4.58	4.7
-Monounsaturated fat,%	1.60	6.8	7.5
-Polyunsaturated fat,%	2.52	4.32	4.66
-Cholesterol,%	-	2.5	2.5
Total Carbohydrate	57.9	65.45	65.45
-Sucrose,%	3.7	11.85	11.85
-Lactose,%	2.01	11.00	11.00
-Fructose,%	0.30	25.15^b^	25.15^b^
Protein,%	28.6	14.3	14.3
Sodium cholate,%	-	1	1
Choline chloride,%	-	0.2	0.2
Thiouracil,%	-	0.075	0.075

^a^ Laboratory Rodent Diet 5001, LabDiet, St. louis, MO, United States.

^b^ Fructose was given in the drinking water.

Sixty rats were randomly divided into six groups of ten rats each (Figure 1): control group (CTRL): fed for six weeks with standard rodent chow; HF6 group: fed for 6 weeks with the high fat - high fructose (HF) diet; HF6+D group: fed for 6 weeks with the HF diet, and then, the HF diet was replaced for 6 weeks with standard chow; HF6+AVO + D group, fed for 6 weeks with the HF diet, and then, the HF diet was replaced for 6 weeks with standard chow plus avocado oil; HF12 group: fed for 12 weeks with the HF diet; HF12 + AVO group: fed for 12 weeks with the HF diet and avocado oil. The rats were sacrificed by decapitation at the end of these treatments and liver sections were obtained for histological analysis, western blot, and mitochondria isolation.

### 2.2 Histological analyses of liver

Liver sections were fixed in 10% formalin, embedded in paraffin blocks and sectioned 5 μM thick. Staining was done with hematoxylin and eosin (H&E). Histology was read by a single independent pathologist, blinded to the experimental design. Steatosis, hypertrophy, and inflammation was scored according to the criteria reported by Liang et al., 2014.

### 2.3 Biochemical parameters in blood

Rats were fasted for 12 h before sacrifice, after which blood was recollected and serum was obtained by centrifugation. The levels of glucose, triglycerides, and total cholesterol were determined with a VITROS assay (Ortho Clinical Diagnostics, Rochester, NY, United States), according to the manufacturer instructions.

### 2.4 Mitochondria isolation and evaluation of respiration

Mitochondria were isolated by differential centrifugation as previously described (García-Berumen et al., 2019). Oxygen consumption rate was determined in freshly isolated mitochondria in the basal state (state 4) and phosphorylating state (state 3) with a Clark-type electrode coupled to a YSI 5300 biological oxygen monitor, connected to a computer for data acquisition. 0.5 mg/ml of mitochondrial protein was placed in a sealed glass chamber containing a respiratory buffer with 100 mM KCl, 75 mM mannitol, 25 mM sucrose and 0.05 mM EDTA (pH 7.4). Final volume was 2.5 ml. Respiration traces were started by adding 10 mM glutamate/malate as respiratory substrate for complex I. Oxygen consumption was monitored for 80 s and then 0.2 mM ADP was added to stimulate state three respiration. 1.4 μg/ml oligomycin was added 220 s later to induce state four respiration and oxygen consumption was further analyzed for 150 s.

### 2.5 Determination of the activities of the ETC complexes

Mitochondria were frozen and thawed twice for determinations of complex I activity. For determinations of complexes II to IV, mitochondria were solubilized with detergent (Ortiz-Avila et al., 2013). Activities were evaluated spectrophotometrically using adequate substrate and inhibitors for each complex. The protocols reported by Ortiz-Avila et al. were used to assess the activity of the complex I (Ortiz-Avila et al., 2015a), complex II and complex II - III (Ortiz-Avila et al., 2013). Complex IV activity was evaluated by the protocol described in (Cortés-Rojo et al., 2009).

### 2.6 Measurement of ROS levels

ROS generation was determined with the fluorescent ROS probe 2′,7′-dichlorodihydrofluorescein diacetate (H_2_DCF-DA). 0.5 mg/ml intact mitochondria and 1.25 mM H_2_DCFDA were incubated in a buffer containing 100 mM KCl, 10 mM HEPES, 3 mM MgCl_2_, and 3 mM KH_2_PO_4_ (pH 7.4) during 20 min at 4 ^∘^C under constant shaking. This was done to allow the uptake of H_2_DCF-DA by mitochondria, which is cleaved into dichlorodihydrofluorescein (H_2_DCFD) by mitochondrial esterases. Then, this suspension was placed in a quartz cuvette and basal fluorescence was recorded. After 1 min, 10 mM glutamate/malate was added as a substrate for complex I and changes in H_2_DCFD fluorescence due to its oxidation by ROS were followed for 15 min. Fluorescence was measured in a Shimadzu RF-5301PC spectrofluorometer (λ_ex_ 491 nm; λ_em_ 518 nm).

### 2.7 Lipid peroxidation assay

This assay was carried out in 0.3 mg/ml of mitochondrial protein by measuring the levels of thiobarbituric acid reactive substances (TBARS), according to the protocol of Buege and Aust (Buege & Aust. 1978). Mitochondrial pellets were washed twice with 50 mM KH_2_PO_4_ buffer (pH 7.4) before TBARS assay to prevent false positives due to the presence of carbohydrates in mitochondrial isolation medium. Absorbance was measured at 532 nm in a Shimadzu UV-2550 UV-VIS spectrophotometer. Data were expressed as μmoles TBARS/mg protein.

### 2.8 Differential spectra of cytochromes

Reduced - minus - oxidized spectra of cytochromes *c* + *c*_1_ and *b* was assessed in a Shimadzu UV2550 double beam spectrophotometer. 1.0 mg/ml intact mitochondria were placed in two cuvettes at a final volume of 2 ml 50 mM KH_2_PO_4_ buffer (pH 7.4). A baseline was recorded with both cuvettes between 500 and 580 nm. Then, the sample cuvette was incubated for 5 min with 10 mM succinate to reduce cytochromes, and 0.75 mM KCN to block cytochrome reoxidation by complex IV. The reference cuvette was oxidized with air. Both cuvettes were placed in the spectrophotometer and spectra were scanned between 500 and 580 nm.

### 2.9 Western blot analysis

Total protein was extracted by sonication of liver homogenates for determinations of cytokines. Protein levels were determined by the Lowry method (Waterborg, 2009). Equal amounts of protein samples were separated in 10% and 12% polyacrylamide gels and transferred to PVDF membranes. Membranes were blocked with 5% skimmed milk for 3 h at 4°C. The membranes were incubated with one of the following primary antibodies: β-actin (sc-1615), TNFα (sc-12744). These antibodies were obtained from Santa Cruz Biotechnology Inc. (Dallas, TX, United States), and diluted to 1:2000. Antibody for IL-6 (ab6672) was obtained from Abcam (Cambridge, United Kingdom) and diluted to 1:4000. After three washes with TBS-T, the membranes were incubated at 4°C for 3 h with 1:2000 donkey anti-rabbit IgG-HRP (sc-2305) and m-IgGκ BP-HRP (sc-516102), obtained from Santa Cruz Biotechnology Inc. (Dallas, TX, United States). Finally, the images were obtained in a multifunctional gel imaging system (Bio-Rad Laboratories, Hercules, CA, United States). The densities of protein bands were quantified using the ImageJ software (Schneider et al., 2012).

### 2.10 Data analysis

The results were expressed as the mean ± standard error of at least three independent experiments, using samples from different animals for each experiment. Statistical differences of the data (*p* < 0.05) were determined by ANOVA, followed by a *post hoc* analysis using the Holm-Šídák test using Sigma Plot v11.0 (Inpixon, Palo Alto, CA, United States).

## 3 Results

### 3.1 Effects of avocado oil on body weight, serum biochemical parameters, and liver histology of rats with NAFLD

**FIGURE 2:**
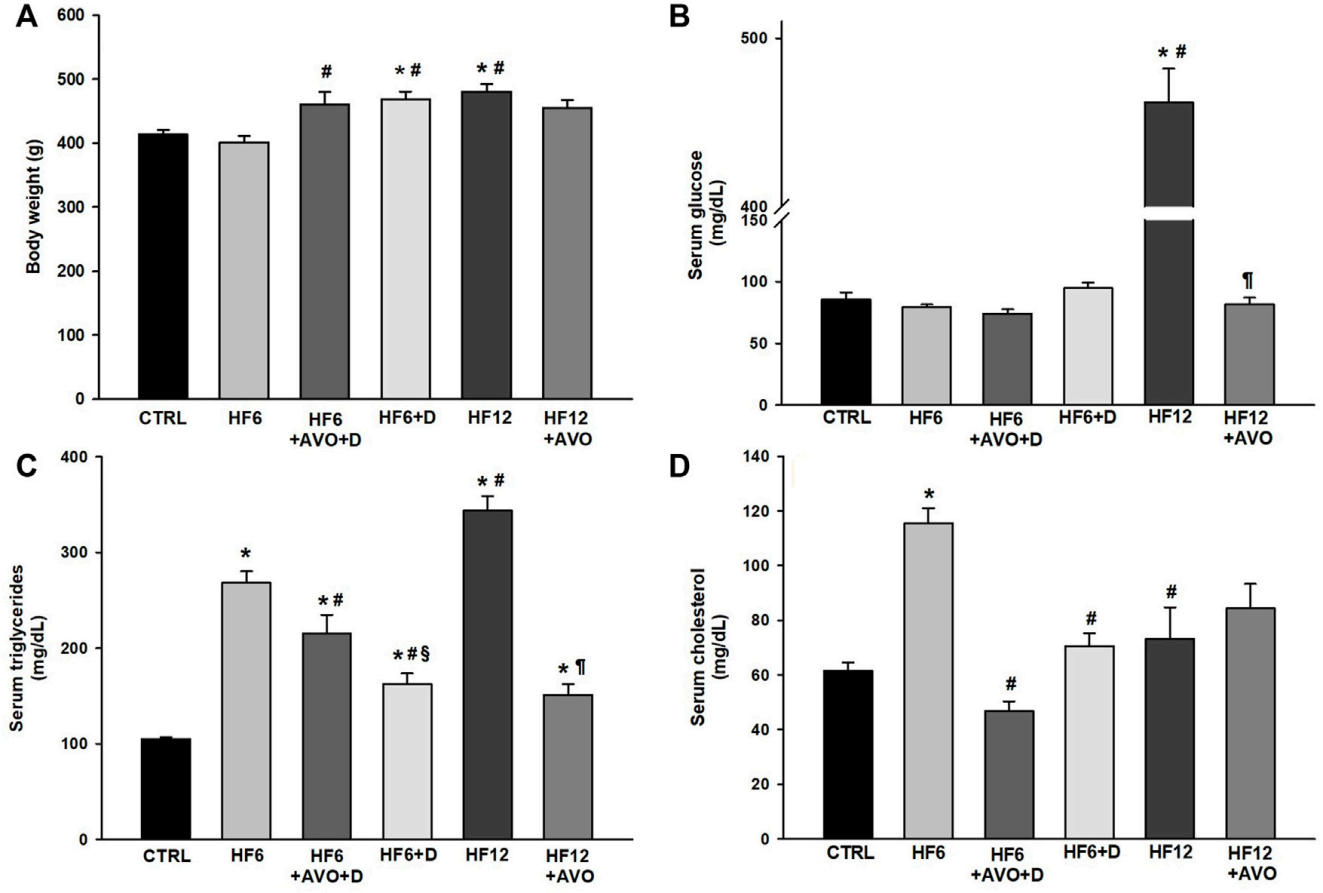
Effects of avocado oil on body weight **(A)**, blood levels of glucose **(B)**, triglycerides **(C)**, and cholesterol **(D)**. Data are expressed as the mean ± standard error of n ≥ 4. ^*^ *p* < 0.05 vs. CTRL; ^#^ *p* < 0.05 vs. HF6; ^§^ *p* < 0.05 vs. HF6+AVO + D; ^¶^ *p* < 0.05 vs. HF12 (ANOVA, Holm-Šídák’s *post hoc* test).

At the end of the treatments, the rats of the HF6+D and HF12 groups weighed ∼61 g more than the rats of the CTRL group. On the other hand, the rats of the HF6 group weighed ∼68 g less than the rats of the HF6+AVO + D, HF6+D, and HF12 groups (Figure 2A). No differences were detected between the HF12 and HF12 + AVO groups.

No changes in glucose levels were observed in all the groups in comparison to the CTRL group (Figure 2B), except by the HF12 group, which displayed hyperglycemia of 461.8 mg/dl. Triglyceride levels were higher in all the groups with respect to the CTRL group to a varying degree (Figure 2C). In comparison to the HF6 group, triglyceride levels decreased in the HF6+AVO + D and HF6+D groups. The HF12 + AVO group had lower triglyceride levels than the HF12 group. Regarding serum cholesterol, this parameter was higher in the HF6 group than in the CTRL group (Figure 2D). Cholesterol levels decreased in the HF6+AVO + D and HF6+D groups with respect to the HF6 group. No statistically significant differences were observed in the HF12 and HF12 + AVO groups versus the CTRL group.

**FIGURE 3:**
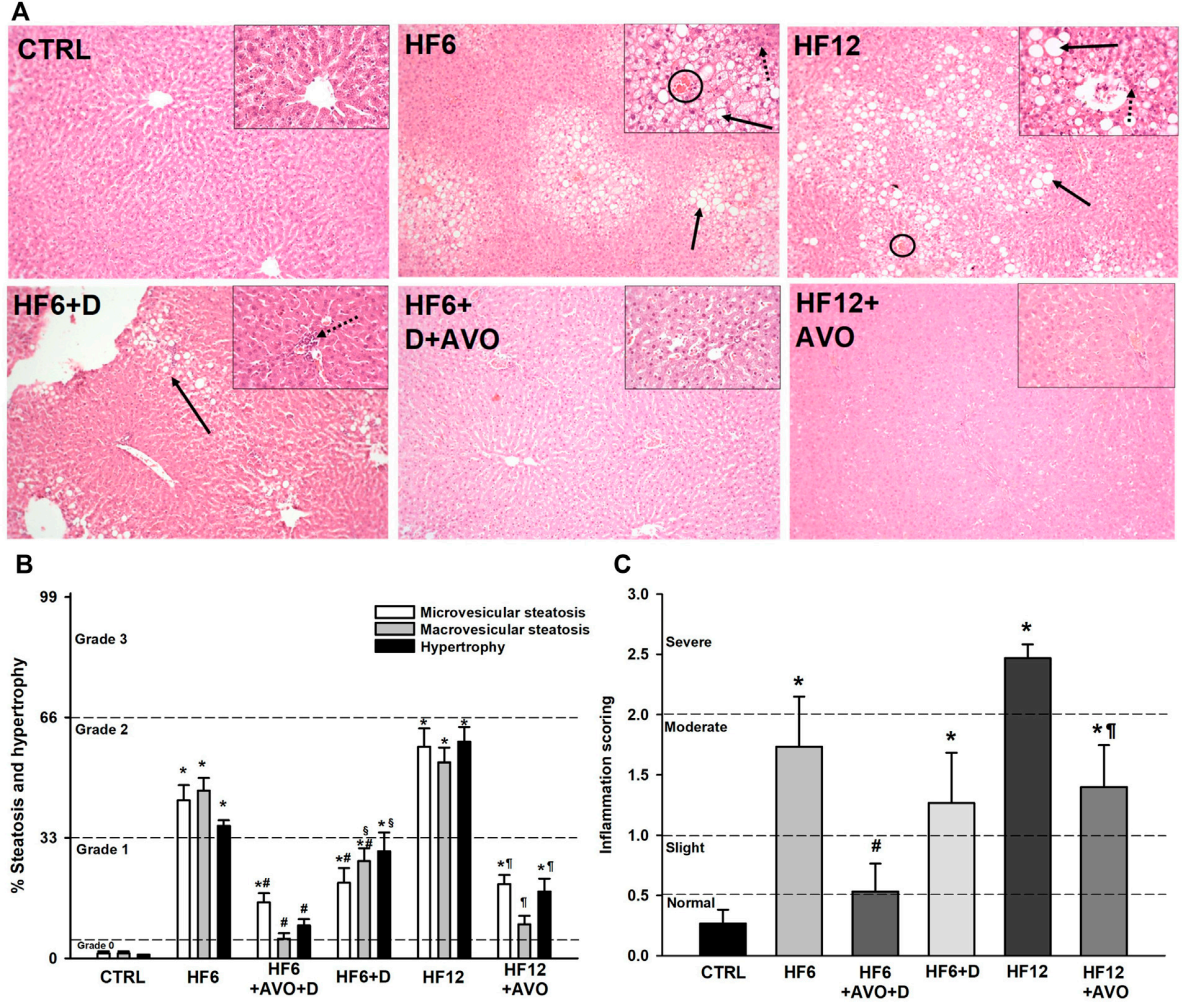
Effects of avocado oil on NAFLD development. The images are representative of four independent hematoxylin and eosin histological examinations of livers dissected from different animals. Black arrows indicate the presence of steatosis; dotted black arrows indicate the presence of inflammatory infiltrates; circles indicate the presence of Mallory’s hyaline. The images were taken at ×10 magnification, while the images in the inserts were taken at ×20 magnification **(A)**. Scoring of the percentage of steatosis and hypertrophy. The dotted lines delimit the percentage of steatosis or hypertrophy corresponding to different categories according to Liang et al. (2014): 0 (<5%), 1 (5%–33%), 2 (34%–66%) and 3 (>66%) **(B)**. Scoring of inflammation: normal (<0.5), slight (0.5–1.0), moderate (1.0–2.0), severe (>2.0). These categories are delimited by dotted lines **(C)**, according to the criteria by Liang et al. (2014). Data are expressed as the mean ± standard error of n = 3. ^*^ *p* < 0.05 vs. CTRL; ^#^ *p* < 0.05 vs. HF6; ^§^ *p* < 0.05 vs. HF6+AVO + D; ^¶^ *p* < 0.05 vs. HF12 (ANOVA, Holm-Šídák’s *post hoc* test).

Liver histology (Figure 3A) shows no histological alterations like steatosis, hypertrophy (Figure 3B) or inflammation (Figure 3C) in the control group. Grade 2 steatosis and hypertrophy was detected in the HF6 and HF12 groups, with the latter group almost reaching grade 3 steatosis and hypertrophy. These alterations were partially reverted with avocado oil, as microvesicular steatosis and hypertrophy were reduced to grade 1 in the HF6+AVO + D group, while macrovesicular steatosis was practically normalized. A similar trend was observed in the HF12 + AVO group when compared to the HF12 group, although macrovesicular steatosis remained in grade 1. Liver alterations were less improved in the HF6+D group in comparison to the HF6+AVO + D group, since both macrovesicular steatosis and hypertrophy were significantly higher in the former group, being in the threshold of grade 2. Inflammation was severe and moderate in the HF12 and HF6 groups, respectively (Figure 3C). Avocado oil improved inflammation, as this parameter decreased from severe in the HF12 group to moderate in the HF12 + AVO group. Likewise, inflammation decreased from moderate in the group HF6 group to normal in the HF6+AVO + D. In contrast, inflammation remained moderate in the HF6+D group, showing no statistically significant differences in comparison to the HF6 group.

### 3.2 Effects of avocado oil on liver mitochondrial function in rats with NAFLD

**FIGURE 4:**
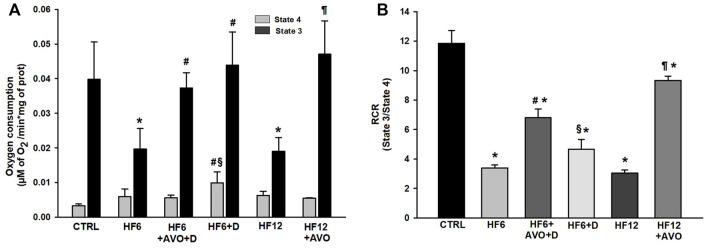
Effects of avocado oil on respiration **(A)** and respiratory control ratio (RCR) **(B)** Data are expressed as the mean ± standard error of n ≥ 4. ^*^ *p* < 0.05 vs. CTRL; ^#^ *p* < 0.05 vs. HF6; ^§^ *p* < 0.05 vs. HF6+AVO + D; *p* < 0.05 vs. HF12 (ANOVA, Holm-Šídák’s *post hoc* test).

The rate of respiration coupled to ADP phosphorylation (state three respiration) decreased by 50% in mitochondria of the HF6 and HF12 groups with respect to mitochondria from the CTRL group (Figure 4A). This effect was prevented in the HF6+AVO + D, HF6+D and HF12 + AVO groups. Respiratory control ratio (RCR) in mitochondria of the HF6 and HF12 groups decreased ∼75% in contrast to the CTRL group (Figure 4B). The RCR in mitochondria of the HF6+AVO + D group was twice higher than the RCR of the HF6 group, while the RCR of the HF12 + AVO group was ∼ 3-fold higher than in the HF12 group. No significant differences were observed between the HF6 and the HF6+D groups. Moreover, the RCR was lower in the HF6+D group than in the HF6+AVO + D group.

**FIGURE 5:**
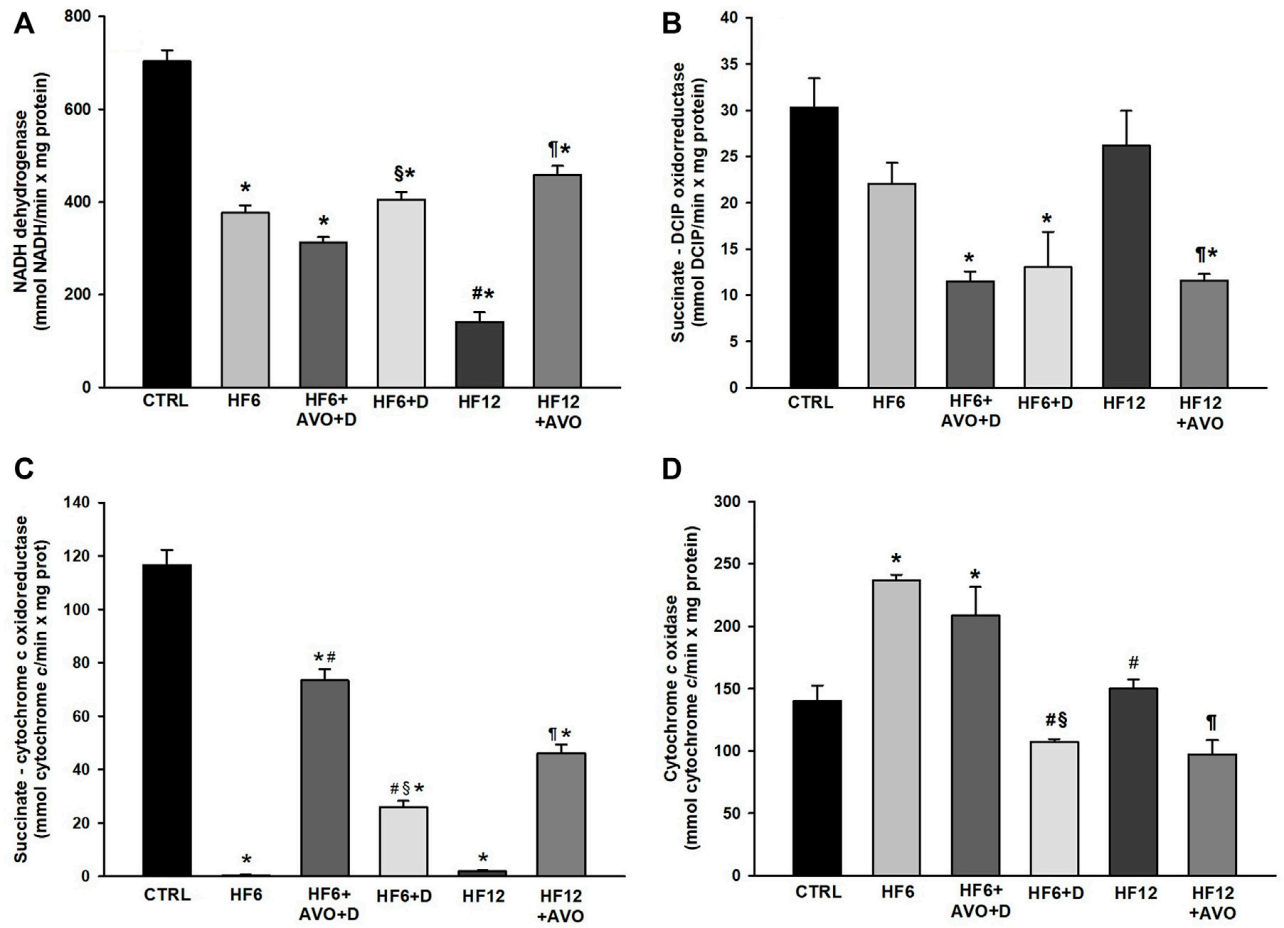
Effects of avocado oil (AVO) on the activities of the complexes I **(A)**, II **(B)**, II - III **(C)**, and IV **(D)** of liver mitochondria from rats fed with a high fat - high fructose diet (HF). Data are expressed as the mean ± standard error of n ≥ 3.^*^ *p* < 0.05 vs. CTRL; ^#^ *p* < 0.05 vs. HF6; ^§^ *p* < 0.05 vs. HF6+AVO + D; *p* < 0.05 vs. HF12 (ANOVA, Holm-Šídák’s *post hoc* test).

Decreased activities of the complex I (Figure 5A) and complex II–III (Figure 5C) were observed in the HF6 and HF12 groups, while non-significant changes were detected in complex II activity (Figure 5B). In contrast, an increment in complex IV activity was observed in the HF6 group, and no changes were observed in the HF12 group (Figure 5D). Regarding the effects of avocado oil, no differences were observed in complex I activity between the HF6 and HF6+AVO + D groups (Figure 5A). In contrast, complex I activity was threefold higher in the HF12 + AVO group than in the HF12 group. No significant differences were observed between the HF6+D and HF6 groups (Figure 5A). The complex II - III activity (Figure 5C) increased in the HF6+AVO + D and HF12 + AVO groups in comparison to the HF6 and HF12 groups, respectively. The complex II - III activity also increased in the HF6+D group in comparison with the HF6 group, without reaching the level of the HF6+AVO + D group (Figure 5C).

The complex II activity decreased ∼60% in the HF6+AVO + D and HF6+D groups in comparison to the CTRL group. Likewise, complex II activity decreased 62% and 56% in the HF12 + AVO group in comparison to the CTRL and HF12 groups, respectively (Figure 5B). Complex IV activity did not decrease in none of the experimental groups with respect to the CTRL group (Figure 5D). Indeed, complex IV activity increased in the HF6 and HF6+AVO + D groups with respect to the CTRL group.

**FIGURE 6:**
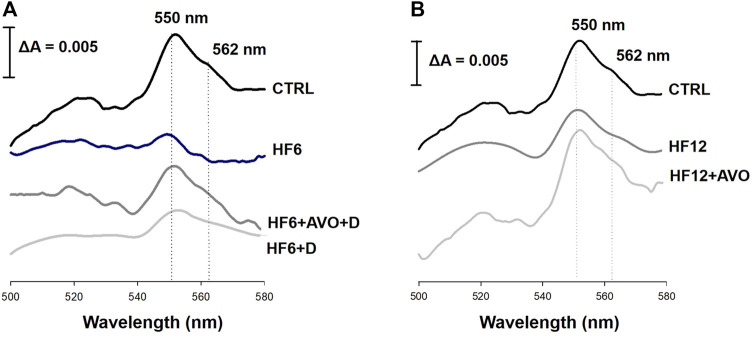
Effects of avocado oil on cytochrome spectra of liver mitochondria. Panel **(A)** spectra from liver mitochondria of the control (CTRL), HF6, HF6+D and HF6+AVO + D groups. Panel **(B)** spectra from liver mitochondria of the CTRL, HF12 group and the HF12 + AVO groups. Succinate was used as a substrate for the reduction of cytochromes. The spectra are representative of four independent determinations carried out with mitochondrial samples obtained from different animals of each group.

The spectrum of cytochromes in the CTRL group shows a peak at 550 nm, corresponding to the reduction of hemes *c* + *c*_1_, and a shoulder at 562 nm, corresponding to the reduction of heme *b* (Figure 6A). A remarkable decrease in the peak at 550 nm is observed in the spectrum of the HF6 group, while the signal at 562 nm is negligible, revealing an impairment in electron transfer through the heme groups of the complex III. Decreased signals of the heme groups were also observed in the mitochondria from the HF12 group (Figure 6B), although this was not as drastic as in the HF6 group. The heme signals in the spectrum of the HF6+AVO + D group were higher than in the spectrum of the HF6 group (Figure 6A). Likewise, the heme signals in the HF12 + AVO group were higher than in the HF12 group (Figure 6B). On the contrary, the HF6+D group did not show a substantial improvement of heme signals (Figure 6A).

### 3.3 Effects of avocado oil on oxidative stress in liver mitochondria from rats with NAFLD

**FIGURE 7:**
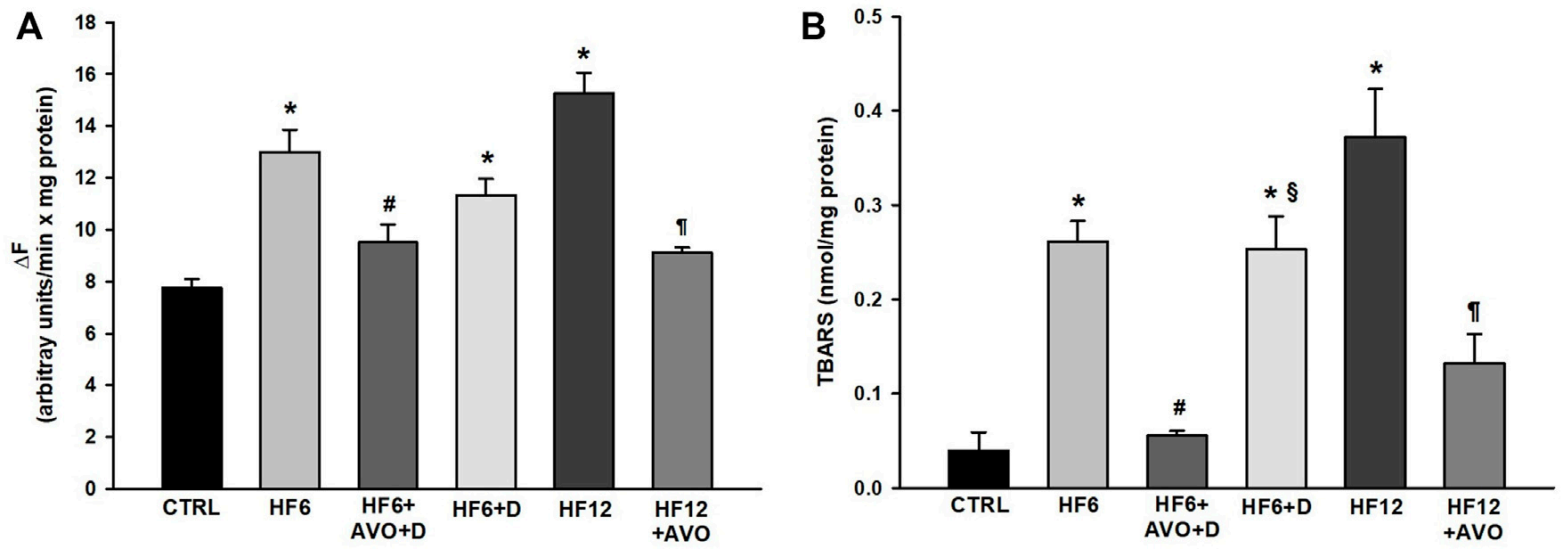
Effects of avocado oil on the levels of ROS **(A)** and lipid peroxidation **(B)** in liver mitochondria. Data are expressed as the mean ± standard error of n ≥ 3.^*^ *p* < 0.05 vs. CTRL; ^#^ *p* < 0.05 vs. HF6; ^§^ *p* < 0.05 vs. HF6+AVO + D; *p* < 0.05 vs. HF12 (ANOVA, Holm-Šídák’s *post hoc* test).

In comparison to the CTRL group, mitochondrial ROS produced with a complex I substrate (Figure 7A) increased 67.8% and 96% in the HF6 and HF12 groups, respectively. ROS decreased 27% and 40% in the HF6+AVO + D and HF12 + AVO groups in comparison to the HF6 and the HF12 groups, respectively. There were no differences between the HF6 and HF6+D groups.

Mitochondrial lipid peroxidation increased 6.6–and 9.4–fold in the HF6 and HF12 groups, respectively, in comparison to the CTRL group (Figure 7B). Lipid peroxidation decreased in the HF6+AVO + D group to the levels of the CTRL group. This parameter also decreased 64% in the HF12 + AVO group in comparison to the HF12 group. No improvement was observed in the HF6+D group in comparison to the HF6 group.

### 3.4 Effects of avocado oil on the levels of pro-inflammatory cytokines in liver of rats with NAFLD

**FIGURE 8:**
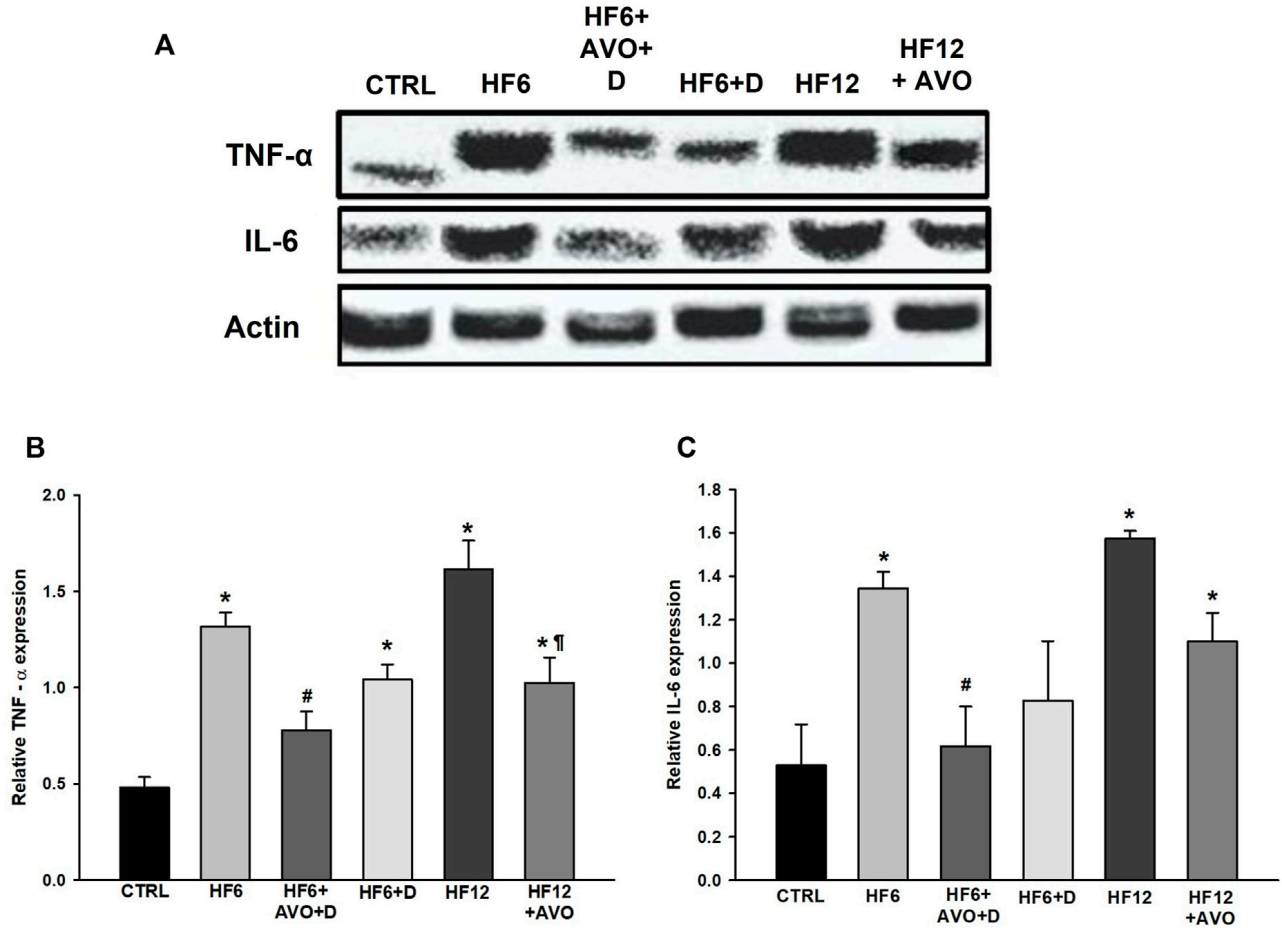
Effects of avocado oil on the levels of proinflammatory cytokines in rat liver. **(A)** Representative blots showing the levels of tumor necrosis factor alpha (TNF-α) and interleukin-6 (IL-6). Quantitative analysis of TNF-α **(B)** and IL-6 **(C)** levels. Data are expressed as the mean ± standard error of n ≥ 3.^*^ *p* < 0.05 vs. CTRL; ^#^ *p* < 0.05 vs. HF6; ^§^ *p* < 0.05 vs. HF6+AVO + D; *p* < 0.05 vs. HF12 (ANOVA, Holm-Šídák’s *post hoc* test).

The levels of interleukin-6 (IL-6, Figures 8A,B) and tumor necrosis factor - alpha (TNF-α, Figures 8A,C) increased more than twice in the HF6 and HF12 groups in comparison to the CTRL group. IL-6 levels were downregulated in the HF6+AVO + D group with respect to the HF6 group. TNF-α levels decreased in the HF12 + AVO and HF6+AVO groups with respect to the levels of the HF12 and HF6 groups, respectively. These cytokines did not decrease in a statistically significant way in the HF6+D group in comparison to the HF6 group.

## 4 Discussion

Hypertriglyceridemia and hypercholesterolemia decreased in the HF12 + AVO (Figure 2D) and HF6+AVO + D (Figure 2C) groups, respectively. These data were expected given avocado oil is an important source of oleic acid (Flores et al., 2019), which improves lipid homeostasis (Rumsey, et al., 1995; Kotake et al., 2004). This is in line with the hypolipidemic effect of avocado oil observed by us in type I diabetic rats (Ortiz-Avila, et al., 2015a), and the beneficial effects of MUFA on the blood lipid profile in humans (Shramko et al., 2020). Severe hyperglycemia was observed in the HF12 group (Figure 2B). This agrees with NAFLD development (Figure 3), since NAFLD contributes to hepatic and whole-body insulin resistance (Akie, et al., 2015). The improvement of NAFLD in the HF12 + AVO group is consistent with normalization of glucose levels in this group, suggesting that avocado oil improved insulin sensitivity. This agrees with the hypoglycemic effect of avocado oil on type-2 diabetes (Ortiz-Avila et al., 2017), and the improvement in glucose homeostasis in rats supplemented with sucrose and avocado oil (Del Toro-Equihua, et al., 2016).

The decrease in electron transfer in the cytochromes of the complex III in the HF6 and HF12 groups (Figure 6) may be the underlying defect in mitochondrial function that contributes to NAFLD development. Accordingly, the HF6 and HF12 groups exhibited negligible levels of complex II - III activity (Figure 5C), the lower rates of respiration in state 3 (Figure 4A), the higher levels of ROS (Figure 7A) and lipid peroxidation (Figure 7B), and the worst outcomes in liver histology (Figure 3). On the contrary, all these alterations and the defective reduction of cytochromes were counteracted by avocado oil in the HF6+AVO + D and HF12 + AVO groups. The normalization of the respiration in state 3 (Figure 4A) and the diminution of steatosis by avocado oil (Figure 3) is consistent with a study showing that the enhancement in state three mitochondrial respiration improves liver steatosis (Akie, et al., 2015). Moreover, the central role of complex III in NAFLD development agrees with the inhibition in complex III activity observed in liver mitochondria from mice with NAFLD (Lee et al., 2018).

The maintenance of the inner mitochondrial membrane integrity is crucial for the function of the ETC complexes as there are specific interactions of cardiolipin and phosphatidylethanolamine with certain subunits of the complexes allowing the correct assembly and catalytic activity of these enzymes. Thus, membrane lipid peroxidation disrupts the organization of the ETC, leading to electron leak and ROS production (Paradies et al., 2019). The higher levels of lipid peroxidation in the HF6, HF6+D, and HF12 groups (Figure 7B) fits well with the prominent decrease in complex II—complex III activity and diminution of cytochromes *c* + *c*_1_ and *b* in these groups (Figures 5C, 6, respectively). Previously, we have shown in yeast mitochondria that lipid peroxidation is directly responsible for impaired electron transfer to cytochrome *b* in the complex III given the hydrophobic character of this subunit (Cortés-Rojo et al., 2009). Moreover, the interaction of cytochrome *c* with cardiolipin in the presence of ROS induces cytochrome *c* detachment from the inner mitochondrial membrane to initiate apoptosis (Wilkinson et al., 2021). Accordingly, mitochondrial ROS production was higher in the HF6, HF6+D, and HF12 groups (Figure 7A). On this basis, it can be proposed that both the HF6 and HF12 regimes disrupt mitochondrial bioenergetics by increasing lipid peroxidation, leading to impairment in electron transfer in the quinone reductase site of the complex III. This causes electron leak and ROS overproduction contributing to the diminution of cytochrome *c*, and overall, to deficient oxidative phosphorylation as revealed by Figure 4. On the contrary, the decrease in lipid peroxidation in both the HF6+AVO + D and HF12 + AVO groups (Figure 7B) led to a better preservation of cytochrome *b* levels (Figure 6) and enhanced electron transfer in the complex II - complex III segment of the ETC (Figure 5C). This decreases ROS production (Figure 7A) and inhibits cytochrome *c* loss (Figure 6). Unexpectedly, in comparison to the control group, complex IV activity increased almost twofold in mitochondria of the HF6 group and remained unchanged in the HF12 group (Figure 5D). In agreement with this, an increase in complex IV activity has been also observed in rats with NAFLD induced by methionine and choline deficiency and it was hypothesized that complex IV enhancement is an adaptive response to increase energy wastage in order to enhance fatty acid oxidation and limit ROS production (Romestaing et al., 2008). Therefore, it may be hypothesized that this adaptation decreases at longer times of exposure to the HF diet, as suggested by the decrease in complex IV activity in the HF12 group in comparison to the HF6 group (Figure 5D).

The RCR reflects the capacity of mitochondria for ATP turnover and substrate oxidation (Brand & Nicholls., 2011). The mitochondria of the HF6 and HF12 groups displayed the lower RCR values (Figure 4B), indicating that NAFLD impairs mitochondrial ATP synthesis, besides decreasing the ability for substrate oxidation (Figures 4A, 5A). This is in concordance with lower ATP levels and decreased F_1_F_0_-ATP synthase activity observed in mice with NAFLD induced by a high fat diet (Lee et al., 2018).

ROS and lipid peroxidation promotes inflammation in NAFLD (Begriche et al., 2006). ROS induces inflammation by stimulating the secretion of TNF-α (Montecucco, et al., 2008). TNF-α in turn induces the actions of IL-6 (Selzner, et al., 2003), which is involved in steatohepatitis development by sensitizing the liver to injury and promoting apoptosis in hepatocytes (Braunersreuther et al., 2012). Therefore, it can be suggested that mitochondrial ROS production in the HF6 and HF12 groups (Figure 7A) led to augmented synthesis of TNF-α (Figure 8C) and IL-6 (Figure 8B), increasing the levels of inflammation in these groups (Figures 3A,C). Thus, the amelioration of liver inflammation in the HF6+AVO + D and HF12 + AVO groups (Figures 3A,C) involves the decrease in mitochondrial ROS production (Figure 7A) and diminution in the synthesis of TNF-α (Figure 8C) and IL-6 (Figure 8B).

On the other hand, the HF6+D group was included in this study to discard that the beneficial effects of avocado oil observed in the HF6+AVO + D group was explained by the substitution of the HF diet by the standard rodent chow rather than by the inclusion of avocado oil. The HF6+D diet counteracted steatosis in a lower degree than the HF6+AVO + D regime (Figure 3B), while had no effect in both hypertrophy (Figure 3B) and inflammation (Figure 3C), improved in a lower degree than the HF6+AVO + D regime the impairments in oxidative phosphorylation (Figure 4B), complex II—complex III activity (Figure 5C), cytochromes reduction (Figure 6A), and the increase in ROS, lipid peroxidation (Figure 7), and TNF-α levels (Figure 8). Thus, the efficacy of the HF6+AVO + D regime is attributable more to the inclusion of avocado oil rather than by the simple substitution of a high fat—high fructose diet by the standard rodent diet. Moreover, the findings in the HF6+AVO + D group shows that avocado oil may be useful in already established NAFLD, which should be the most expected scenario in real-life settings since NAFLD has been an overlooked - underdiagnosed disease. This is because NAFLD has been historically viewed as a benign disease, of little concern, with questionable clinical relevance, despite mortality by liver disease occurring in large cohorts of diabetic patients (Ratziu et al., 2015).

Avocado oil contains multiple bioactive molecules including oleic acid, its main fatty acid, carotenoids, phytosterols, chlorophylls and tocopherols (Ashton et al., 2006; Berasategi, et al., 2012). β-sitosterol is the more abundant phytosterol in avocado oil (Berasategi, et al., 2012), and it may be responsible for a great part of the effects seen in this study, as it decreases blood glucose levels, improves insulin sensitivity and liver steatosis in diabetic rats consuming a high fat and sucrose diet (Babu and Jayaraman, 2020), diminishes oxidative stress by augmenting glutathione content (Vivancos and Moreno, 2005) and enhances ETC function (Wong et al., 2016). However, a link between the effects of β-sitosterol on NAFLD, glucose homeostasis and mitochondrial function remains to be elucidated. We recognize that a main limitation of this work is that the molecule(s) responsible for the effects of avocado oil were not identified. However, the notable effects of avocado oil on all the alterations elicited by the HF diet guarantee further research to establish avocado oil as a potential source of molecules for the pharmacologic treatment of metabolic syndrome.

In conclusion, avocado oil improved NAFLD induced by a high fat - high fructose diet by improving mitochondrial function, leading to lower levels of ROS, lipid peroxidation, proinflammatory cytokines, and inflammation. All these benefits were accompanied by improvements in dyslipidemia and hyperglycemia. Thus, avocado oil is a promising source of molecules for the treatment of metabolic syndrome and its associated morbidities by targeting mitochondrial dysfunction and oxidative stress.

## Data Availability

The raw data supporting the conclusions of this article will be made available by the authors, without undue reservation.
